# Molecular Biomarkers for Prediction of Targeted Therapy Response in Metastatic Breast Cancer: Trick or Treat?

**DOI:** 10.3390/ijms18010085

**Published:** 2017-01-04

**Authors:** Angela Toss, Marta Venturelli, Chiara Peterle, Federico Piacentini, Stefano Cascinu, Laura Cortesi

**Affiliations:** Department of Oncology and Hematology, University Hospital of Modena and Reggio Emilia, via del Pozzo 71, 41121 Modena, Italy; martaventurelli@msn.com (M.V.); chiara.peterle@gmail.com (C.P.); federico.piacentini@unimore.it (F.P.); stefano.cascinu@unimore.it (S.C.); hbc@unimore.it (L.C.)

**Keywords:** breast cancer, molecular biomarker, targeted therapy, treatment resistance

## Abstract

In recent years, the study of genomic alterations and protein expression involved in the pathways of breast cancer carcinogenesis has provided an increasing number of targets for drugs development in the setting of metastatic breast cancer (i.e., trastuzumab, everolimus, palbociclib, etc.) significantly improving the prognosis of this disease. These drugs target specific molecular abnormalities that confer a survival advantage to cancer cells. On these bases, emerging evidence from clinical trials provided increasing proof that the genetic landscape of any tumor may dictate its sensitivity or resistance profile to specific agents and some studies have already showed that tumors treated with therapies matched with their molecular alterations obtain higher objective response rates and longer survival. Predictive molecular biomarkers may optimize the selection of effective therapies, thus reducing treatment costs and side effects. This review offers an overview of the main molecular pathways involved in breast carcinogenesis, the targeted therapies developed to inhibit these pathways, the principal mechanisms of resistance and, finally, the molecular biomarkers that, to date, are demonstrated in clinical trials to predict response/resistance to targeted treatments in metastatic breast cancer.

## 1. Introduction

Breast cancer (BC) represents the most common cancer among women worldwide, with an estimated incidence of 246,660 new cases (29% of all sites cancers) and 40,450 estimated deaths (14% of all sites) in 2016 in the United States [1]. Despite the relevant progress in prevention, diagnosis, and treatment of BC and the consequent improvement in overall survival, metastatic BC continues to be an incurable disease with a median survival time of 18–24 months, depending on the extension of the tumor and its histopathological and molecular profile [2,3,4,5,6].

At the molecular level, BC is a heterogeneous disease that develops and progresses from alterations that take place in the genes that govern cell growth, proliferation and differentiation [7,8]. In the last two decades, the increasing knowledge on genomic abnormalities associated with gain of function or downstream signal activation involved in the BC evolution, allowed to find new therapeutic approaches “tailored” on the molecular alteration identified. The revolutionary era of targeted therapy shifted the classic paradigm of BC treatment from a “stratified oncology” based on pathological and clinical parameters [9] to a “personalized medicine” based on the match between the targeted drug and the molecular alteration that confers to cancer cells a survival advantage [10]. Currently, an increasing number of these molecularly targeted drugs is available for clinical practice or in the context of clinical trials and, nowadays, the main challenge remains the identification of predictive biomarkers for the selection of the optimal treatment, in order to spare patients from the side effects associated with treatment and to minimize the overall cost [11].

This review aims to reassume the main molecular pathways involved in BC carcinogenesis, the targeted therapies developed to inhibit those pathways, the principal mechanisms of resistance and, finally, the molecular biomarkers that, to date, have demonstrated to predict response/resistance to targeted treatments.

## 2. Signaling Pathways Involved in Breast Cancer Carcinogenesis

A large body of evidence in literature has already pointed out that cancer is the result of subsequent genetic mutations in somatic cells [12,13]. These mutations affect and activate a number of cellular pathways, which are responsible for growth, proliferation and differentiation in BC cells [14,15].

### 2.1. Estrogen Signaling Pathway (Figure 1)

Steroid hormones contribute to carcinogenesis in BC acting on cell growth, development, differentiation, and homeostasis. Estrogens activate both nuclear estrogen receptors (ERα and ERβ, genomic pathway) and membrane estrogen receptors (mER, non-genomic pathway) [16]. In the genomic pathways, ligand-activated nuclear ER dimerizes and translocates in the nucleus where it binds to DNA to regulate the activity of different genes. On the other hand, in the non-genomic pathway, the mER activates a variety of signal transduction pathways, including the MAPK pathway and the PI3K/AKT/mTOR pathway [17].

### 2.2. Epidermal Growth Factor Receptors (HER) Family (Figure 1)

The epidermal growth factor receptor (HER) family includes four different receptors: erbB1 (HER1 or EGFR), erbB2 (HER2), erbB3 (HER3), and erbB4 (HER4). This family of tyrosine kinase receptors regulates several biological processes and is particularly involved in cell proliferation control, differentiation, and survival [18]. A high expression of HER2 has been detected in 20% to 30% of human breast carcinomas and correlates with a worse prognosis, since it is associated with higher aggressiveness, shorter survival and high risk of endocrine therapy resistance [19]. In general, these receptors are composed of an extracellular domain for ligand binding, a transmembrane segment, and an intracellular domain with tyrosine kinase activity. The ligand binding causes conformational changes in the receptor that allows dimerization (homo or hetero-dimerization) with the other Epidermal Growth Factor Receptors and induces intracellular kinase domains phosphorylation with the activation of “downstream” signaling pathways, including PI3K/AKT/mTOR, MAPK, and JAK/STAT pathways, which promote proliferation and survival [20,21].

### 2.3. PI3K/AKT/mTOR Pathway (Figure 1)

PI3K/AKT/mTOR pathway is one of the main downstream pathways involved in cancer cell proliferation and is activated by several receptor tyrosine kinases (RTKs), such as EGFR, IGF-1, FGFR, MET, etc. PI3K represents a family of kinases classifiable into four main classes. Class I PI3Ks have a catalytic subunit known as p110, with four isoforms: p110 α (encoded by *PIK3CA*), p110 β (encoded by *PIK3CB*), p110 γ (encoded by *PIK3CG*), and p110 Δ (encoded by *PIK3CD*) [22]. This first class of PI3Ks is the one mainly involved in oncogenesis and has been the target for anti-cancer drug development. The principal role of Class I PI3Ks is to phosphorylate the phoshatidyl-inositol(4,5)P2 (PIP2) to phospha-tidilinositol(3,4,5)P3 (PIP3) [22]. After phosphorylation, PIP3 activates AKT. Activated AKT recognizes a wide range of substrates, with their activating or inhibiting functions, such as mTOR, NF-κB (nuclear factor of κB), MDM2 (a negative regulator of the oncosoppressor p53), GSK3β (involved in cell cycle and glucose metabolism processes), etc. Therefore, activated AKT mediates and regulates different biological processes, including growth independence, apoptosis and proliferation [23,24].

PTEN (phosphatase and tensin homolog) is the negative regulator of PI3K/AKT/mTOR pathway due to its dephosphorylating action. PTEN is a tumor suppressor with diverse functions, including regulation of cell cycle, apoptosis and metastasis [25,26]. Mutations, or a reduced expression of the *PTEN* gene, are associated with a wide variety of human tumors, including breast cancer [27]. Somatic mutations in all points of this pathway have been identified in BC. Particularly, mutations of *PIK3CA* have been found in almost 30% of all sporadic BC [28] with a wide range of frequencies among BC subtypes [29,30], whereas the frequency of *PTEN* loss is 30%–40% and the somatic intragenic *PTEN* mutation frequency is <5% [31].

### 2.4. MAPK Signaling Pathway (Figure 1)

MAPK may lead to an uncontrolled cell cycle, resistance to apoptosis and to chemotherapy, targeted therapies, and radiotherapy. The interaction between the RTKs (such as EGFR, PDGFR, FGFR, etc.) and their ligands allows RAS (a family of small GTPases) to activate the protein kinase activity of RAF, a serine/threonine kinase. RAF kinase, as a cascade, phosphorylates and activates MEK (mitogen-activated protein kinase). MEK (MEK1 and MEK2) phosphorylates and activates a mitogen-activated protein kinase, ERK (extracellular-signal-regulated kinase, also called MAPK), which translocates into the nucleus where triggers several transcription factors that mediate expression of oncogenes involved in proliferation and survival [32,33]. Overall, the most frequent somatic mutations occurring in the MAPK cascade involve *MAP3K1* (8%) and *MAP2K4* (4%) [29].

### 2.5. RB-E2F and p53 Pathways (Figure 2)

Cell cycle regulation can be perturbed by a wide range of mechanisms, including activation of RB-E2F pathway and the p53 pathway. RB is one of the best-known oncosuppressors, responsible for turning on or off the cell cycle [34]. One downstream consequence of RB activation is the inhibition of E2F activity, which is important for the transcription of several genes that are required for progression through the cell cycle. Particularly, E2F up-regulates the cyclin E gene and then, the cyclinE-CDK2 holoenzyme completes the phosphorylation and inactivation of RB [34,35,36]. In addition, the Cyclin D1, upregulated by growth factors like EGF and estrogen, binds to CDK4/6 and partially phosphorylates and inactivates RB [37]. In the p53 pathway, signals such as DNA damage, induce the tumor suppressor ARF (alternate reading frame) to increase p53 levels by sequestering MDM2, which facilitates the degradation and inactivation of p53. Simultaneously, the kinases ATM/ATR phosphorylate p53 directly and through activation of CHK2 or CHK1. Among the p53 target genes are WAF1, an inhibitor of cyclin-dependent protein kinases (CDKs) that, among other activities, causes cell-cycle arrest, and BAX, which promotes apoptotic cell death. RB also regulates p53 activity through a trimeric p53-MDM2-RB complex [38]. Overall, the most frequent somatic mutations occurring in these pathways involve *TP53* (37%) and *RB1* (2%) [29].

### 2.6. Angiogenic Pathway (Figure 1)

Tumor angiogenesis means the growth of new blood vessels, which are needed by the tumor in order to grow [39]. A huge number of molecules are involved in this process, some of them with a facilitating role (pro-angiogenic factors, such as the vascular endothelial growth factor, VEGF), others with an inhibiting role (anti-angiogenic factors). Activation of pro-angiogenic pathways in cancer cells is critical to cancer development [40]. Particularly, signal transduction induced by VEGF involves binding to tyrosine kinase receptors and results in endothelial cell proliferation, migration, and new vessel formation [41].

### 2.7. SRC Pathway (Figure 1)

SRC (Rous Sarcoma) plays a critical role in the development and progression of many solid tumors and is also associated to the development of drug resistance [42]. SRC is the best-known member of a family of non-receptor cytoplasmatic tyrosine kinases (SFKs) involved in regulatory mechanisms of cell proliferation, growth, migration, and other neoplastic features. The SRC activation implicates a cascade of signaling pathways involved in oncogenesis, including PI3K/AKT/mTOR, MAPK, and JAK/STAT [43].

### 2.8. HSP90 Mechanism of Action (Figure 3)

Under stressful conditions, the heat shock protein 90 (HSP90) molecular chaperone protects oncoproteins (such as HER2, AKT, c-MYC, etc.) from degradation via the ubiquitin-proteasome pathway. HSP90 is up-regulated in cancers, and this contributes to increase proliferation and decreased apoptosis [44].

### 2.9. DNA Repair Mechanisms (Figure 4)

Several mechanisms are involved in the repair of DNA damage, which includes single-strand breaks (SSBs) and double-strand breaks (DSBs). The SSB repair is accomplished by the base excision repair (BER), the nucleic acid excision repair (NER) and the mismatch repair (MMR). Poly (ADP-ribose) polymerase (PARP) is an enzyme involved in the BER. DSBs are corrected by the homologous recombination (HR) and non-homologous end joining (NHEJ) systems. When a defect occurs in one of the enzymes involved in HR, such as BRCA1 and BRCA2, the DSBs are repaired from error-prone mechanisms, mostly NHEJ. The NHEJ activation results in increased risk of new chromosomal defects and, thus, the development of cancer [45,46,47,48].

### 2.10. JAK/STAT Pathway (Figure 1)

The interaction between the RTKs or the cytokine receptors and their ligands allows a conformational change in the JAK (Janus Kinase) inactive form, placed on the intracellular tails of the receptor. Active JAKs phosphorilate tyrosine residues of the intracytoplasmatic domain of the receptor itself, creating a binding domain for STAT protein (signal transducer and activator of transcription) that floats around in the cytoplasm. Phosphorylated STAT dimerizes with other STAT proteins and the activated dimer translocates into the nucleus and promotes transcription of genes involved in proliferation, differentiation, and apoptosis processes. Dysregulations in JAK-STAT functionality result in immune disorders and cancers [49].

### 2.11. Immune Pathway (Figure 5)

Immune checkpoints are molecules in the immune system able to either turn up or down an immunogenic signal. Under physiologic conditions, a balance between co-inhibitory and co-stimulatory signals maintains self-tolerance and immune homeostasis, protecting tissues from unnecessary damage. Tumor cells take advantage of these mechanisms to evade immune recognition by inhibiting the T cell signal. For their activation, T cells require two signals: the first signal is initiated by the T cell receptor (TCR) through antigen recognition, whereas the second one is mediated by the interaction between receptors and ligands of co-stimulatory and co-inhibitory signals (the immune checkpoints), which include CTLA-4 (cytotoxic T-lymphocyte-associated protein 4), PD-1 (programmed death 1), and PD-L1 (programmed death-ligand 1). In tumors, the expression of immune inhibitory molecules following oncogenic transformation results in the attenuation of immune reactions, immune resistance and, thus, cancer cell survival [50,51].

## 3. Targeted Therapies for the Treatment of Advanced Breast Cancer

The definition “targeted therapy” includes those treatments that use substances able to identify and attack specific subtypes of cancer cells, with the aim of minimizing damage to normal cells. Some targeted therapies block the action of certain enzymes, proteins, or other molecules involved in the pathways of tumor growth and spread. Other types of targeted therapies enhance the immune system response against cancer cells or kill the cancer cells though the release of toxic substances. Since the early history of targeted therapy, with the advent of endocrine treatment more than 50 years ago, significant progress has occurred in this field.

### 3.1. Endocrine Therapy

Treating BC by hormonal deprivation is an historical observation dating back to 1896, when Beatson described a new treatment strategy for inoperable breast carcinoma by ovaries removal [52]. Since then, a wide range of drugs have been developed with the aim of inhibit the estrogen signaling pathway and treat the hormone receptor (HR) positive breast tumors. Three different categories of endocrine treatment are now available:

- SERMs (selective estrogen receptor modulators): they are competitive partial agonists of the estrogen receptor. Particularly, tamoxifen is the oldest and the most well-known drug of this category [53]. Subsequently, toremifene citrate was developed with the goal of achieving efficacy similar to that of tamoxifen and with an improved safety profile. To date, although studies have not confirmed a better safety profile, clinical data have supported the efficacy and safety of toremifene for the treatment of BC in postmenopausal patients [54].
- Aromatase Inhibitors: they stop the production of estrogen in postmenopausal women by inhibiting the activity of aromatase. The third-generation aromatase inhibitors have largely replaced tamoxifen in the treatment of postmenopausal HR positive BC patients. They are classified into irreversible steroidal inhibitors, such as exemestane, that form a permanent and deactivating bond with the aromatase enzyme, and non-steroidal inhibitors, such as anastrozole and letrozole, that act via reversible competition for the aromatase enzyme [55,56,57].
- SERDs (selective estrogen receptor down-regulators): they reduce ER α protein levels, as well as block estrogen receptor activity degrading and destroying the estrogen receptor. The only approved SERD for the treatment of metastatic HR positive BC is fulvestrant [58,59].

### 3.2. Anti-HER Agents

Since the development of the first anti-HER2 agent, the prognosis of patients with HER2 positive tumors, which represent the 20%–25% of all BC, has significantly improved [60,61]. In the following years, several therapeutic strategies for the treatment of HER2 positive BC have been developed:

- Recombinant humanized monoclonal antibodies (trastuzumab and pertuzumab): binding the extracellular domain of HER2, trastuzumab blocks the dimerization of HER2 while pertuzumab inhibits the heterodimerization of HER2 with other HER receptors, inhibiting the downstream signaling pathways (PI3K and MAPK) with a cytostatic mechanism; they also have a cytotoxic mechanism through the activation of the antibody dependent cell-mediated cytotoxicity (ADCC) [62,63,64].
- Antibody-drug conjugates (TDM1): it conjugates efficacy of trastuzumab with the cytotoxic effect of DM1 (emtansine), a tubulin inhibitor [65,66].
- Receptor tyrosine kinase inhibitors (RTKIs) (lapatinib): they inhibit enzyme function of HER family intracellularly, binding competitively to the intracellular kinase domain ATP-binding site of EGFR and/or HER2 [67].
- Other anti-HER2 compounds are still under evaluation in clinical trials such as HER2 vaccines, other monoclonal antibodies (such as ertumaxomab and margetuximab), and defucosylated trastuzumab [11].

### 3.3. Compounds Targeting PI3K/AKT/mTOR Pathway

The protein kinases involved in this pathway are attractive and promising drug targets for BC treatment, especially in endocrine and anti-HER2 resistance settings [68]. Several molecules have already been investigated and showed interesting results in clinical trials:

AKT phosphorylation inhibitors. MK2206, ridaforolimus, perifosine, and others are currently under evaluation in phase II clinical trials [69,70].

mTOR inhibitors. On the basis of BOLERO2 trial [71], in 2012 everolimus has been approved by the Food and Drug Administration (FDA) for the treatment of postmenopausal women with advanced HR positive, HER2 negative BC in combination with exemestane, after failure of treatment with letrozole or anastrozole. Moreover, everolimus has also been studied in association to chemotherapy and trastuzumab in the HER2 positive setting [72,73].

PI3Kα-selective inhibitors (alpelisib), PI3Kα/δ-selective inhibitors (pictilisib), PanPI3K inhibitors (buparlisib). The first results of the phase III BELLE2 trial showed that the association of buparlisib to fulvestrant is able to improve progression free survival (PFS) compared to fulvestrant alone in patients with locally advanced or metastatic HR positive BC resistant to aromatase inhibitors [74]. On the other hand, the FERGI and the PEGGY phase II trials failed in showing any significant clinical benefit from adding pictilisib to either fulvestrant or paclitaxel in patients with HER2 negative, HR positive BC [75,76]. Finally, a phase II trial showed that the combination of taselisib plus fulvestrant had an acceptable side effect profile and clinical activity in patients with HER2 negative, HR positive advanced BC [77].

### 3.4. Farnesyl Transferase Inhibitors

Farnesyl transferase (FTase) inhibitors are a class of antineoplastic agents developed to specifically inhibit RAS signaling pathway. Particularly, Tipifarnib is a non-peptidomimetic, orally-bioavailable, competitive inhibitor of FTPase that has already shown an activity in preclinical models and in phase I and II studies in association to chemotherapy and endocrine therapy [78,79,80].

### 3.5. Anti-RTKs (FGFR, MET, and IGF-1R)

Several tyrosine kinase inhibitors (TKi) and monoclonal antibodies targeting single or multiple RTKs have been studied:

The anti-FGFR1 dovitinib has shown antitumor activity in advanced BC with FGF pathway alterations, suggesting that FGFR could be a therapeutic target in these patients that warrants further investigation [81]. Moreover, other FGFR inhibitors are currently under investigation in phase I/II trials, such as luvitanib and nintedanib.

In a recent phase II study, the anti-MET tivantinib did not meet pre-specified statistical targets for efficacy in triple-negative BC patients [82]. On the other hand, in heavily pretreated metastatic BC patients, the anti-MET multi-targeted TK inhibitor cabozantinib demonstrated clinical activity, including objective response and disease control [83]. Several other anti-MET compounds are still under investigation in phase II clinical trials, such as foretinib and onartuzumab.

Finally, several IGF-1R inhibitors have been tested in clinical trials but, to date, have failed to show any clinical benefit in unselected patients [84].

### 3.6. Cyclin-Dependent Kinase (CDK) Inhibitors

Palbociclib: the PALOMA1 phase II study revealed an impressive improvement in PFS with palbociclib combined to letrozole [85] thus, in February 2015, FDA approved palbociclib plus letrozole for first line treatment in ER positive HER2 negative advanced or metastatic BC. These data of PFS improvement were then confirmed in the PALOMA 2 phase III study [86]. Subsequently, on the basis of the results of the PALOMA3 trial [87], FDA extended the approval of palbociclib to include therapy in combination with fulvestrant for HR positive, HER negative advanced or metastatic BC after progression during endocrine therapy.

Ribociclib: in the phase III MONALEESA2 study, ribociclib plus letrozole showed significantly longer PFS than placebo plus letrozole in patients receiving initial systemic treatment for HR-positive, HER2-negative advanced breast cancer, but with a significant increase in the rate of toxicity [88]. Moreover, the phase III MONALEESA3 is investigating ribociclib in combination with fulvestrant and the phase III MONALEESA7 trial is evaluating ribociclib in combination with tamoxifen and goserelin or a non-steroidal aromatase inhibitor and goserelin for the treatment of premenopausal women.

Abemaciclib: the phase II MONARCH1 trial, abemaciclib induced objective tumor responses as a monotherapy in patients with refractory HR positive HER2 negative metastatic BC following multiple prior therapies [89]. Moreover, the phase III MONARCH2 study is evaluating the combination of fulvestrant plus abemaciclib and the phase III MONARCH3 study is evaluating anastrozole or letrozole plus abemaciclib in first line treatment.

### 3.7. Angiogenesis Inhibitors

Strategies to inhibit angiogenesis include the use of bevacizumab, a monoclonal antibody targeting VEGF-A, and tyrosine kinase inhibitors (TKIs) (such as sunitinib). These targeted agents have been studied both as monotherapies and in combination with chemotherapeutics. Nevertheless, in several studies including the E2100, AVADO, and RIBBON-1 studies, the combination of angiogenesis inhibitors with standard chemotherapy regimens in metastatic BC has resulted in improvement in PFS, but not in overall survival (OS), while TKIs have not shown any efficacy in BC treatment yet [90,91,92,93,94].

### 3.8. SFK Inhibitors

Targeting SRC family kinases (SFKs) has the ability to inhibit different steps of carcinogenesis. Dasatinib and other antineoplastic agents of this category are ATP-competitive inhibitors of SFKs, with multiple effects still not fully understood [95,96]. Particularly, dasatinib is a tyrosine kinase inhibitor that inhibits multiple oncogenic tyrosine kinases including SFKs, BCR-ABL, PDGF, and c-KIT and that showed a role in osteoclast proliferation, survival, and resorptive function [97]. Nevertheless, in a phase II trial, dasatinib did not demonstrate any effectiveness in controlling bone-predominant metastatic BC in patients unselected by molecular biomarkers [98].

### 3.9. HSP90 Function Inhibitors

HSP90 inhibitors have shown early promising results in defined molecular subgroups of solid tumors, such as the HER2-positive BC [99], and are now under investigation in several clinical trials. To date, promising results have been observed with tanespimycin in combination with trastuzumab in patients progressing on trastuzumab [100] and, with single agent ganetespib in HER2-positive tumors and TNBC [101].

### 3.10. PARP Inhibitors

PARP inhibitors exploit the synthetic lethality concept to prevent the DNA damage repair in cells with homologous recombination deficiency, causing cancer cell death. Clinical evidence of PARP inhibitors efficacy was initially slowed by negative results from a phase III trial of iniparib, a compound at first classified as a PARP inhibitor [102]. After it was shown that iniparib does not inhibit PARP in intact cells, clinical development of PARP inhibitors gained renewed interest. Currently, five compounds are under investigation in clinical trials: olaparib in the phase III OlimpiA and OlimpiAD studies, veliparib in the phase II BROCADE study and two phase III studies, niraparib in the phase III BRAVO study, talazoparib in two phase II studies, and the phase III EMBRACA study and rucaparib in two phase II studies [103,104,105]. Notably, based on the high sensitivity of BRCA-deficient cells to PARP inhibitors, BRCA-mutation carriers are the most appropriate candidates for treatment with PARP inhibitors.

### 3.11. Immunotherapy

Agonists of co-stimulatory receptors or antagonists of inhibitory receptors might lead to an amplification of antigen-specific T cell response against tumor cells [106]. On these bases, multiple immunotherapy approaches are under investigation in patients with BC: vaccines that elicit strong specific immune responses to tumor antigens, such as WT-1 [107], HER2 [108], and NY-ESO-1 [109]; strategies involving adoptive transfer of in vitro-expanded, naturally-arising, or genetically-engineered tumor-specific lymphocytes; therapeutic administration of monoclonal antibodies to target tumor cells; approaches that inhibit the molecular or cellular mediators of cancer-induced immunosuppression, such as CTLA-4 and PD-1 [50]. Particularly, the recent phase Ib KEYNOTE-012 trial demonstrated that the PD-1 inhibitor pembrolizumab has activity and an acceptable toxicity profile as single-agent therapy in heavily pretreated, advanced triple-negative BC [110]. Other clinical trials are currently evaluating nivolumab (anti-PD1), ipilimumab (anti-CTLA-4), atezolizumab (anti-PD-L1), durvalumab (anti-PD-L1), and tremelimumab (anti-CTLA-4) in BC patients.

## 4. Resistance Mechanisms to Targeted Therapies

The incoming resistance to targeted therapies is a major limitation to treatment efficacy. Primary or intrinsic resistance occurs when an inherent feature of the cancer cells prevents the drugs from working. On the other hand, secondary, or acquired, resistance occurs when cancer cells become resistant during treatment and it manifests over time after an initial response [111].

BC behaves as an evolving entity, with metastases acquiring different biological profiles as compared to their matched primary tumors [112,113]. A large body of evidence indicated relevant rates of discordance between primary tumor and subsequent metastatic disease [114,115]. This biological evolution is exacerbated by the selective pressure imposed by treatments during the natural history of the disease, thus modifying its sensitivity or resistance to therapies. On these bases, the importance of molecular re-characterization of metastatic BC has become central in the management of the disease and it has been recently acknowledged in the clinical recommendations of principal international guidelines.

In HR positive BC, multiple mechanisms of endocrine resistance have been described, including mutations in *ESR1* gene which encode ERα. Particularly, mutations in *ESR1* appear to be rare in treatment naive setting and more frequent in advanced BC previously treated with aromatase inhibitors [116]. Other mechanisms of endocrine resistance are: up regulation of alternative crosstalk signaling pathways, altered expression of specific microRNAs, balance of co-regulatory proteins, and genetic polymorphisms involved in endocrine therapy metabolic activity [16]. Interestingly, some of these mechanisms have been exploited with the aim to find strategies able to overcome endocrine treatment resistance:

Hyperactivation of PI3K/AKT/mTOR pathway through mutations/amplifications affecting the genes encoding the PI3K catalytic subunits (*PIK3CA*, *PIK3CB*), PI3K regulatory subunit (*PIK3R1*), receptor tyrosine kinases (HER2, FGFR1), K-Ras, PI3K effectors (AKT1, AKT2, PDK1), and loss of PTEN and INPP4B [117]. On these bases, the mTOR inhibitor everolimus has already been approved for advanced HR positive, HER2 negative BC in combination with exemestane and, several other inhibitors of this pathway in association to endocrine therapy are under evaluation.

Dysregulation of the cell-cycle machinery and activation of cyclin-dependent kinases (CDKs), and particularly CDK4/6 (through the CCND1 amplification, CDK4 amplification, loss of CDKN1B, CDKN2A, and CDKN2B, RB1 mutation) [118]. On these bases, the CDK4/6 inhibitors palbociclib and ribociclib have been investigated in association to endocrine therapy and are now entering clinical practice.

Epigenetic aberrations through the methylation of specific DNA genes (such as *RASSF1A*, *CCND2*, *GSTP1*, and *TWIST*) that are able to alter the expression of ER. On this basis, histone deacetylase (HDAC) inhibitors have been investigated and showed the capability of partially restoring ER expression. Particularly, entinostat demonstrated to restore sensitivity to hormonal therapy and to improve PFS and OS when given in combination with exemestane, in patients with ER-positive advanced BC resistant to previous aromatase inhibitors [119].

In HER2 positive BC, the main mechanisms of resistance to anti-HER2 agents include: impaired access to HER2 by expression of extracellular domain-truncated HER2 (p95 HER2); overexpression of Mucin 4, a mucin protein encoded by the *MUC4* gene that serves as a ligand for HER2; alternative signaling from other RTKs, such as IGF-1R, other HER family members, or MET; loss of downstream controllers (PTEN, p27); and activation of downstream signaling pathways (PI3K/AKT/mTOR and MAPK pathways) [11,120]. The main strategies developed to overcome resistance in trastuzumab-refractory HER2-positive tumors are: TKIs (lapatinib), antibody-drug conjugates (TDM-1), dual inhibition of HER2 (pertuzumab + trastuzumab, lapatinib + trastuzumab), HER2 vaccines, other monoclonal antibodies (margetuximab and ertumaxomab), and defucosylated trastuzumab. Furthermore, since the activation of growth factor receptors (such as IGF-1R, HER, and MET) and ER activate crosstalking downstream signaling pathways, particularly the PI3K/AKT/mTOR and MAPK pathways, some of the strategies developed in order to overcome anti-HER2 treatment resistance overlap to those developed for endocrine-resistant disease. Particularly, PI3K/AKT/mTOR pathway inhibitors, inhibitors of alternative signaling molecules (IGF-1R, FGFR and MET) and HSP90 inhibitors have been evaluated in trastuzumab-refractory HER2-positive tumors.

## 5. Predictive Molecular Biomarkers

Currently, several molecularly targeted drugs are available in clinical practice or in the context of clinical trials and, nowadays, with the aim to personalize treatment strategies, the challenge is mainly represented by the optimal selection of the most effective treatment for each patient. On these bases, parallel to the development of new therapeutic strategies, researchers are looking for molecular biomarkers able to predict response to those treatments. For some of these targeted therapies, predictive biomarkers have already been identified in clinical trials and are described below and in Table 1.

### 5.1. Fulvestrant

A recent prospective-retrospective analysis evaluated circulating tumor DNA (ctDNA) in archived baseline plasma from the SoFEA trial in order to assess the impact of ESR1 mutation on the efficacy of fulvestrant and examestane. ctDNA is tumor-related circulating free DNA released in the blood by tumor cells in necrosis and represents a promising biomarker for non-invasive assessment of tumor DNA. Patients with ESR1 mutations in ctDNA had improved PFS after taking fulvestrant compared with exemestane, whereas patients with wild-type ESR1 had similar PFS after receiving either treatment. In this analysis, the detection of ESR1 mutations in plasma DNA predicted relative resistance to exemestane and relative sensitivity to fulvestrant [121].

### 5.2. Everolimus

A retrospective exploratory biomarkers analysis of the BOLERO2 trial found that PFS benefit with everolimus was maintained regardless of alteration status of *PIK3CA*, *FGFR1*, and *CCND1*, or the pathways of which they are components [128]. An additional analysis studying the ctDNA suggested that patients with *PIK3CA* activating mutations obtain a similar PFS benefit from everolimus compared to patients without *PIK3CA* mutations (*PI3KCA* WT 56.7% PFS 7.36 month HR 0.43; *PI3KCA* mutant 43.3% PFS 6.9 HR 0.37) [129]. Overall, these analyses support the evidence that the efficacy of everolimus is independent of *PIK3CA* mutational status. Additionally, an exploratory analysis of the BOLERO3 trial showed greater benefit derived from the addition of everolimus in HER2 positive BC patients with a low PTEN concentration than in those with a high PTEN concentration and in patients with a high pS6 concentration than in those with low pS6 concentration, while again *PIK3CA* mutations did not seem to predict any benefit [127]. On the other hand, the combined analysis of BOLERO1 and 3 showed a significant PFS benefit in patients with HER2 positive advanced BC having tumors with with *PIK3CA* mutations, *PTEN* loss or hyperactive PI3K pathway (*PIK3CA* mutations and/or *PTEN* loss and/or *AKT1* mutation) treated with everolimus in combination with trastuzumab, plus either paclitaxel or vinorelbine. On the contrary, an everolimus benefit was not seen in patients with wild-type *PIK3CA*, normal *PTEN*, or normal PI3K pathway activity [123].

Another recent analysis of BOLERO2 evaluated the incidence of *ERS1* mutation and its clinical impact. The result of this analysis demonstrated that addition of everolimus was associated with improved PFS for patients with wild-type or D538G mutation but not for those with Y537S mutation. Overall, this analysis showed a potential lack of benefit in tumors with either the Y537S mutation alone or with both Y537S and D538G [122].

Finally, an exploratory analysis of the TAMRAD study evaluated potential predictive markers of everolimus efficacy using primary tumor samples obtained from enrolled patients. Particularly, the analysis evaluated the proteins that result in mTORC1 activation, the *PIK3CA* gene and the *KRAS* gene. The patients most likely to have an improvement in TTP with tamoxifen/everolimus therapy, compared with tamoxifen alone, were those with high p4EBP1, low 4EBP1, low liver kinase B1, low pAKT, and low PI3K [125].

### 5.3. Buparlisib

A sub-analysis of the BELLE2 study indicated that patients who had mutant *PIK3CA* detected in their ctDNA had much better outcomes if they received buparlisib plus fulvestrant when compared with those who received fulvestrant alone (HR 0.56; *p*-value < 0.001). This study suggests that the research of mutational status of *PI3KCA* in ctDNA may help in the selection of patients who benefit the most from adding a PI3K inhibitor to endocrine therapy [74].

Moreover, the BELLE-3 phase III trial is investigating the efficacy of buparlisib plus fulvestrant in postmenopausal patients with HR+/HER2− advanced BC previously treated with an aromatase inhibitor and refractory to mTOR inhibitor-based therapy. Also in this study, PFS, OS, ORR, and the clinical benefit rate will be evaluated based on ctDNA *PIK3CA* mutation status [130].

### 5.4. Pictilisib

In the FERGI phase II trial, the *PIK3CA* mutation status was not associated to an improvement in PFS or ORR with pictilisib [75]. In addition, also the PEGGY phase II trial failed in showing any significant clinical benefit from adding pictilisib to paclitaxel in patients with advanced HER2 negative, HR positive BC, in either the ITT population or the *PIK3CA* mutated subgroup [76].

### 5.5. Alpelisib and Taselisib

Two ongoing phase III trials are evaluating the use of *PIK3CA* mutation in ctDNA as a predictor marker for a response to the α-specific PI3K inhibitor: SOLAR-1 and SANDIPIPER. The first trial investigates the combination of alpelisib and fulvestrant, the patients are screened and stratified based on *PI3KCA* mutation status and randomized to receive fulvestrant in combination with either alpelisib or placebo [131,132]. The second study follows a phase II trial that showed how the combination of taselisib plus fulvestrant had an acceptable side effect profile and clinical activity in patients with HER2 negative, HR positive advanced BC, with a numerically higher response in patients with *PIK3CA* mutations [77]. SANDIPIPER studies the combination of taselisib plus fulvestrant in patients with ER positive, HER2 negative locally advanced or metastatic BC enriched for patients with *PIK3CA* mutant tumors [133].

### 5.6. Dovitinib

Dovitinib has already shown antitumor activity in FGFR-amplified BC cell lines in preclinical models, and in a phase II trial recruiting patients with metastatic BC with and without FGFR1 amplification, dovitinib showed increased activity in BC with FGF pathway amplification. Particularly, dovitinib showed more potent antitumor activity in patients with FGF pathway-amplified BC (amplifications in FGFR1, FGFR2, or FGF3) [81]. On these bases, dovitinib is under evaluation in combination with fulvestrant in a phase II randomized trial in patients with BC who have FGF-pathway amplifications (FGFR1, FGFR2, or FGF3) [134].

### 5.7. Trastuzumab

HER2 overexpression is predictive for anti-HER2 therapy and its research is already routine in clinical practice [60,135].

### 5.8. Pertuzumab

In the CLEOPATRA trial, pertuzumab in association to trastuzumab and docetaxel showed to improve PFS and OS in the first line treatment of HER2 positive metastatic BC. Baselga et al. performed a biomarker analysis of tumor samples from patients in CLEOPATRA but, similarly to prior studies, identified prognostic and no predictive markers. Particularly, high HER2 protein, high HER2 and HER3 mRNA levels, wild-type *PIK3CA* and low serum HER extracellular domain were associated with a significantly better prognosis [64]. At the moment HER2 remain the only marker suited for patient selection for trastuzumab plus pertuzumab-based regimen in HER2 positive metastatic BC.

### 5.9. LAPATINIB and TDM-1

Lapatinib has been the only HER2 target agent available for trastuzumab resistant patients, until the superiority of TDM-1 over lapatinib and capecitabine was demonstrated by the results of the EMILIA trial in 2012. In a sub analysis of the EMILIA trial, tumors tissues were evaluated for HER2, EGFR, and HER3 mRNA expression by quantitative reverse transcriptase PCR, for PTEN protein expression by IHC, and for *PIK3CA* mutations using a mutation detection kit. The presence of *PIK3CA* mutations was associated with shorter PFS and OS durations in patients who received lapatinib, but did not adversely affect efficacy with T-DM1. Moreover, T-DM1 appeared to result in a greater PFS benefit versus lapatinib also in patients with absent or decreased PTEN expression. Additionally, consistently with previous reports [136,137,138], the authors observed that tumors with HER2 mRNA over-expression (over the median level) were characterized by increased sensitivity to treatment with either T-DM1 or lapatinib. On the other hand, the PFS and OS benefit with T-DM1 was greater in patients with tumors expressing EGFR or HER3 mRNA below the median level [124]. These results confirm the previous preclinical findings that loss of *PTEN* or activation of *PIK3CA* through hotspot mutations (E545K in the HD in exon 9 and H1047R in KD exon 20) confer resistance to lapatinib in HER2 overexpressing BC cells [139].

### 5.10. Neratinib

Approximately 1.6% of all newly-diagnosed BC may harbor a *HER2* mutation, and most of these patients do not have *HER2* gene amplification or overexpression. This percentage might be even higher for patients who have relapsed. These *HER2* somatic mutations are an alternative mechanism to activate HER2 in BC. *HER2* mutation positive patients represent a subpopulation that likely benefits from HER2-targeted drugs, particularly irreversible inhibitors such as neratinib. Interestingly, in preclinical models all the 13 functionally characterized mutations were sensitive to neratinib, including those that cause resistance to lapatinib [140,141]. The preliminary analysis from the SUMMIT phase II study demonstrated encouraging sign of clinical activity of neratinib in patients with heavily pre-treated, *HER2* mutant, HER2 non-amplified metastatic BC [142].

### 5.11. Palbociclib

An exploratory analysis of the PALOMA1 trial revealed no additional predictive value of CCND1 amplification or p16 loss for palbociclib efficacy [85,143]. Moreover, in the PALOMA3 study *PIK3CA* status in cfDNA did not show to significantly affect neither the magnitude of benefit associated with fulvestrant plus palbociclib nor the hormone-receptor status of BC [87].

A recent prospective retrospective analysis performed ctDNA analysis in archived baseline plasma from SoFEA and PALOMA3 trials in order to assess the impact of *ESR1* mutation on the efficacy of current therapies. In the PALOMA3 trial, *ESR1* mutations were associated with acquired resistance to prior aromatase inhibitors. Nevertheless, fulvestrant plus palbociclib improved PFS compared with fulvestrant plus placebo in both *ESR1* mutant and *ESR1* wild-type patients [121].

### 5.12. Bevacizumab

The AVADO trial showed that high baseline plasma VEGF-A and VEGFR-2 concentrations were associated with greater PFS benefit from bevacizumab [126].

### 5.13. Entinostat

The phase II Encore 301 study showed that the addition of the HDAC inhibitor entinostat to exemestane improve PFS and OS in patients with advanced ER positive BC failing an aromatase inhibitor [119]. The biomarker analysis showed that patients who presented hyperacetylation of lysines induced by HDAC inhibitors in blood samples had a reduced risk of disease progression. An ongoing confirmatory phase III trial E2112 will better define the role of HDAC inhibitors and confirm the change in protein lysine acetylation as biomarker of response [144].

### 5.14. Parp-Inhibitors

Predictive biomarkers of response to PARP inhibitors have yet to be identified. A candidate biomarker is the combination of three DNA-based homologous recombination deficiency (HRD) scores. These HRD scores highly correlated with defects in *BRCA1/2*, and are associated with a response to platinum therapy in triple-negative BC [145]. The value of an HRD score in predicting a response to PARP inhibitors is currently under evaluation in prospective studies with advanced BC patients.

### 5.15. Immunotherapy

PD-L1 expression on tumor cells may not be considered a definitive predictive biomarker for the response to PD-1/PD-L1 blockade, since in some tumors the response to PD-1/PD-L1 blockades is independent of PD-L1 expression [146]. On the other hand, PD-L1 expression seems to correlate with the presence of tumor-infiltrating lymphocytes (TILs) and TILs showed to possess the possibility to predict the response of checkpoint blockades in 41 patients with melanoma, non-small cell lung carcinoma, renal cell carcinoma, colorectal carcinoma, or castration-resistant prostate cancer [147]. Nonetheless, further research is needed to accurately identify patients who will benefit from checkpoint blockades.

## 6. Conclusions

Recent evidence emerging from clinical trials provided demonstrations that the genetic landscape of any given tumor is able to dictate its sensitivity or resistance profile to matched anticancer agents and some studies have already showed that in patients receiving therapies matched with their molecular alterations, the objective response rate may be higher and the PFS and survival may be longer [148,149,150]. Nevertheless, these results are still widely debated [151] and, to date, very few predictive molecular biomarkers have been identified for the treatment decision-making in metastatic BC patients.

A promising field of research for the detection of predictive biomarkers is represented by the study of microRNA (miRNA). The miRNAs are endogenous, small non-coding RNA molecules that showed an aberrant expression in breast cancer patients [152]. Particularly, miRNA dysregulation, in either cancer tissues or plasma, may predict a patient’s response to treatments. For instance, up-regulated miR-210 in tissues has been associated with higher risk of recurrence in tamoxifen-treated patients [153], while increased levels of miR-210 in the plasma were correlated with trastuzumab resistance [154]. However, the study of miRNA requires further research and an optimization of detection strategies, therefore, to date, this approach has not yet been introduced in clinical practice.

In the era of personalized medicine, future research should be directed into two parallel directions. On the one hand, the increasing knowledge on cancer signaling pathways should encourage the identification of new molecular targets for the development of anti-cancer agents that are likely to improve treatment response and circumvent resistance. On the other hand, more translational research is required to identify biomarkers that could help to predict response and resistance, in order to improve the selection of the optimal targeted treatment for each patient.

## Figures and Tables

**Figure 1:**
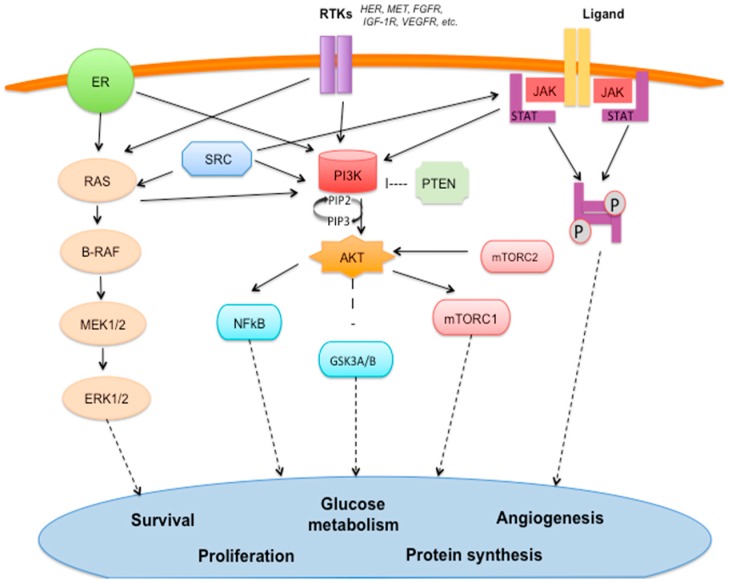
The crosstalking network of signaling pathways involved in breast cancer development and progression: estrogen receptor (ER) signaling pathway, receptor tyrosine kinase (RTK) pathway, PIK3/AKT/mTOR pathway, MAPK signaling pathway, angiogenic pathway, SRC pathway and JAK/STAT pathway. ER: estrogen receptor, RAS: rat sarcoma viral oncogene homolog, B-RAF: murine sarcoma viral oncogene homolog, MEK1/2: MAPK/Erk kinase 1/2, ERK1/2: extracellular-signal-regulated kinase 1/2, SRC: rous sarcoma, RTKs: receptor tyrosine kinases, JAK: Janus kinase, STAT: Signal Transducer and Activator of Transcription, PI3K: Phosphoinositide 3-kinase, PTEN: Phosphatase and tensin homolog, AKT: protein kinase B, NFκB: nuclear factor κ-light-chain-enhancer of activated B cells, GSK3A/B: Glycogen synthase kinase-3 α/β, mTORC1/2: mammalian target of rapamycin complex 1/2, PIP: phosphatidylinositol phosphate, P: phosphorylated. Solid arrow: activation. Dashed arrow: activation of nuclear transcription factors. T-bar: inhibition.

**Figure 2:**
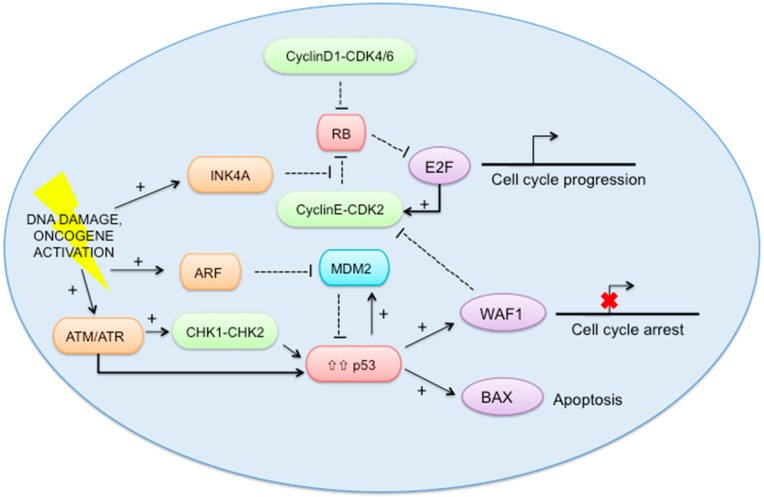
The RB-E2F and p53 pathways. INK4A: p16 protein, RB: retinoblastoma, E2F: E2 factor, CDK: cyclin dependent kinase, ARF: p14 protein, MDM2: Mouse double minute 2 homolog, ATM: ataxia-telangiectasia mutated, ATR: ATM- and Rad3-Related, CHK1: cell cycle checkpoint kinase 1, CHK2: cell cycle checkpoint kinase 2, P53: tumor suppressor P53, WAF1: cyclin-dependent kinase inhibitor 1, BAX: BCL2 Associated X. Arrow with +: activation. T-bar: inhibition.

**Figure 3:**
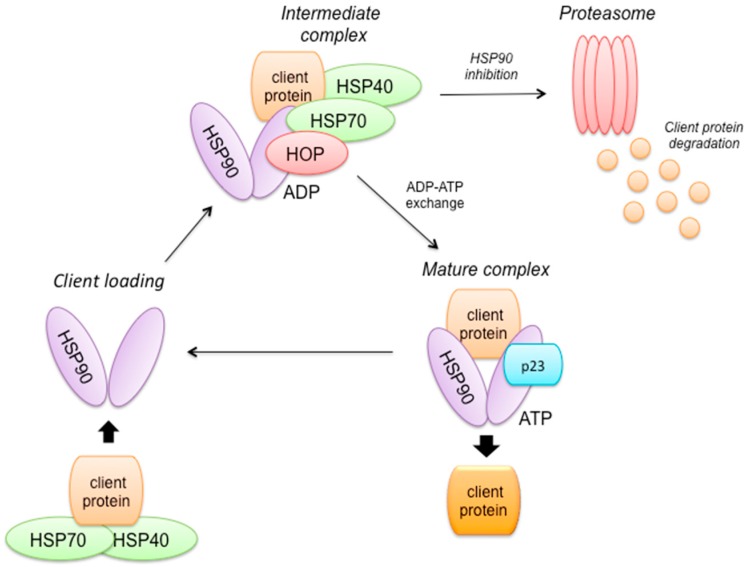
HSP90 mechanism of action. The binding of a client protein to HSP90 requires the co-operation of another chaperone (HSP70 and its co-factor HSP40). HOP mediates interaction between HSP70 and HSP90. The exchange of ADP to ATP induces dissociation of HSP70 and its co-chaperones from the complex that associate then with p23, forming a mature complex. Under stressful conditions, HSP90 protects oncoproteins (such as HER2, AKT, c-MYC, etc.) from degradation. HSP90: heat shock protein 90 kDa, HSP70: Heat-shock protein of 70-kDa, HSP40: heat shock protein 40 kDA, HOP: Hsp organizing protein, ADP: Adenosine diphosphate, ATP: Adenosine triphosphate.

**Figure 4:**
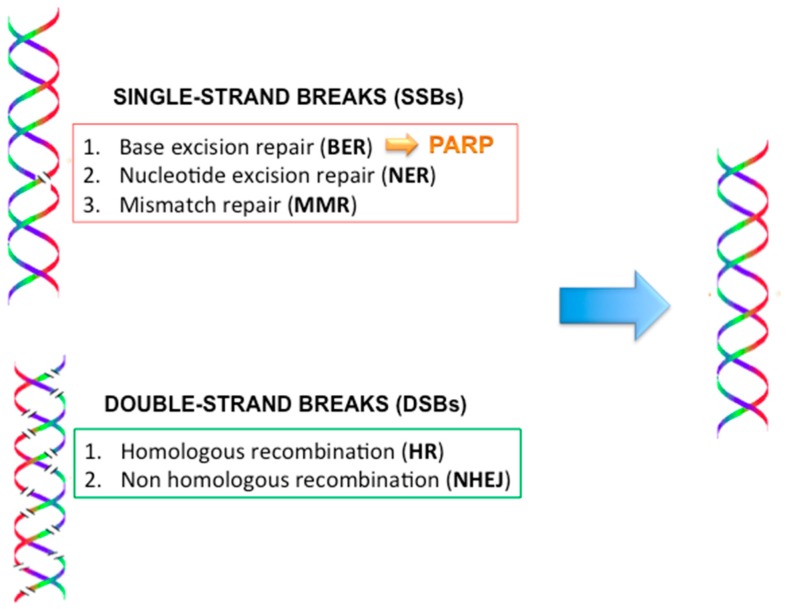
DNA repair mechanisms and the role of PARP enzymes. PARP: Poly (ADP-ribose) polymerase.

**Figure 5:**
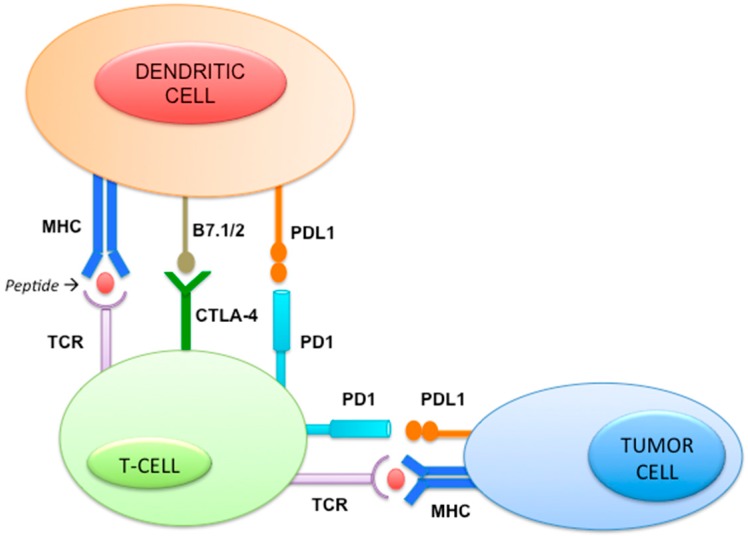
Immune pathway and the immune-checkpoints. TCR: T cell receptor, CTLA4: T-lymphocyte-associated antigen 4, B7.1: Cluster of differentiation 80, B7.2: Cluster of differentiation 86, MHC: Major histocompatibility complex, PD1: programmed cell death protein 1, PDL: PD1 ligand.

**Table 1:** Molecular biomarkers predictive of response to targeted treatment in clinical trials.

Molecular Biomarkers	Method of Analysis	Targeted Therapy	References
ESR1 mutations	ctDNA	sensitivity to FULVESTRANT	Fribbens 2016; [121]
	ctDNA	resistance to EXEMESTANE	Fribbens 2016; [121]
	Y537S mutation in ctDNA	resistance to EVEROLIMUS	Chandarlapaty 2016; [122]
PIK3CA mutations	Tumor tissue	sensitivity to EVEROLIMUS	André 2016; [123]
	ctDNA	sensitivity to BUPARLISIB	Baselga 2015; [74]
	Tumor tissue	sensitivity to TASELISIB	Dickler 2016; [89]
	Tumor tissue	resistance to LAPATINIB	Baselga 2016; [124]
AKT1 mutations	Tumor tissue	sensitivity to EVEROLIMUS	André 2016; [123]
mTORC1 activation (high p4EBP1, low 4EBP1, low liver kinase B1, low pAkt, and low PI3K)	Tumor tissue	sensitivity to EVEROLIMUS	Treilleux 2015; [125]
FGF pathway amplified	Tumor tissue	sensitivity to DOVITINIB	André 2013; [81]
HER2 amplification	Tumor tissue	sensitivity to TRASTUZUMAB	Dawood 2010; [60]
	Serum samples and tumor tissue	sensitivity to PERTUZUMAB	Baselga 2014; [64]
	Tumor tissue	sensitivity to LAPATINIB	Baselga 2016; [124]
	Tumor tissue	sensitivity to TDM1	Baselga 2016; [124]
EGFR down expression	Tumor tissue	sensitivity to TDM1	Baselga 2016; [124]
HER3 down expression	Tumor tissue	sensitivity to TDM1	Baselga 2016; [124]
VEGF-A and VEGFR-2 high concentration	Serum samples	sensitivity to BEVACIZUMAB	Miles 2013; [126]
Low PTEN concentration	Tumor tissue	sensitivity to EVEROLIMUS	Jerusalem 2013; [127] André 2016; [123]
		sensitivity to TDM1	Baselga 2016; [124]
High pS6 concentration	Tumor tissue	sensitivity to EVEROLIMUS	Jerusalem 2013; [127]
Hyperacetylation of lysines	Serum samples	sensitivity to ENTINOSTAT	Yardley 2013; [119]
